# Human Long Noncoding RNA Regulation of Stem Cell Potency and Differentiation

**DOI:** 10.1155/2017/6374504

**Published:** 2017-08-30

**Authors:** Seahyoung Lee, Hyang-Hee Seo, Chang Youn Lee, Jiyun Lee, Sunhye Shin, Sang Woo Kim, Soyeon Lim, Ki-Chul Hwang

**Affiliations:** ^1^Institute for Biomedical Convergence, College of Medicine, Catholic Kwandong University, Gangneung-si, Gangwon-do, Republic of Korea; ^2^Brain Korea 21 PLUS Project for Medical Science, Yonsei University, Seoul, Republic of Korea; ^3^Department of Integrated Omics for Biomedical Sciences, Yonsei University, Seoul, Republic of Korea

## Abstract

Because of their capability of differentiation into lineage-specific cells, stem cells are an attractive therapeutic modality in regenerative medicine. To develop an effective stem cell-based therapeutic strategy with predictable results, deeper understanding of the underlying molecular mechanisms of stem cell differentiation and/or pluripotency maintenance is required. Thus, reviewing the key factors involved in the transcriptional and epigenetic regulation of stem cell differentiation and maintenance is important. Accumulating data indicate that long noncoding RNAs (lncRNAs) mediate numerous biological processes, including stem cell differentiation and maintenance. Here, we review recent findings on the human lncRNA regulation of stem cell potency and differentiation. Although the clinical implication of these lncRNAs is only beginning to be elucidated, it is anticipated that lncRNAs will become important therapeutic targets in the near future.

## 1. Introduction

Stem cells are specialized cells capable of differentiating into lineage-specific cells. Depending on their potential to differentiate and source of origin, stem cells can be broadly categorized into embryonic stem cells (ESCs), induced pluripotent stem cells (iPSCs), and adult stem cells such as bone marrow mesenchymal stem cells (BM-MSCs) and adipose-derived stem cells (ADSCs). Due to their differentiation and self-renewal abilities, stem cells have been highly regarded as an effective therapeutic modality in regenerative medicine [1]. Effective stem cell therapeutics should be based on a meticulously designed strategy, especially if the goal of stem cell-based therapy involves in situ differentiation of stem cells. In other words, to develop an effective stem cell-based therapeutic strategy with predictable results, deeper understanding of the underlying molecular mechanisms of stem cell differentiation and/or pluripotency maintenance is required. Therefore, it is worth reviewing the key factors involved in the transcriptional and epigenetic regulation of stem cell differentiation and maintenance. One of such key factors of stem cell biology is a group of noncoding RNAs (ncRNAs) [2, 3].

A large portion of the human genome is transcribed into RNAs without coding proteins. Noncoding RNAs are such RNAs that are not translated into proteins. Based on their sizes, they can be classified into the following 2 categories: small ncRNAs and long ncRNAs. Small ncRNAs refer to ncRNAs shorter than 200 nucleotides long, while long ncRNAs (lncRNAs) refer to ncRNAs composed of 200 or more nucleotides. Small ncRNAs can be further categorized into subcategories based on their length, function, and subcellular localization such as microRNAs (miRNAs), short interfering RNAs (siRNAs), Piwi-interacting RNAs (piRNAs), small nucleolar RNAs (snoRNAs), short hairpin RNAs (shRNA), and other short RNAs [4, 5]. These small ncRNAs have been implicated in stem cell biology, and many excellent articles on the role of these small ncRNAs in stem cell biology are already available [6–8]. Therefore, only the lncRNAs with documented functions affecting human stem cell biology will be discussed in this particular review.

## 2. General Regulatory Mechanisms of lncRNA

**Figure 1:**
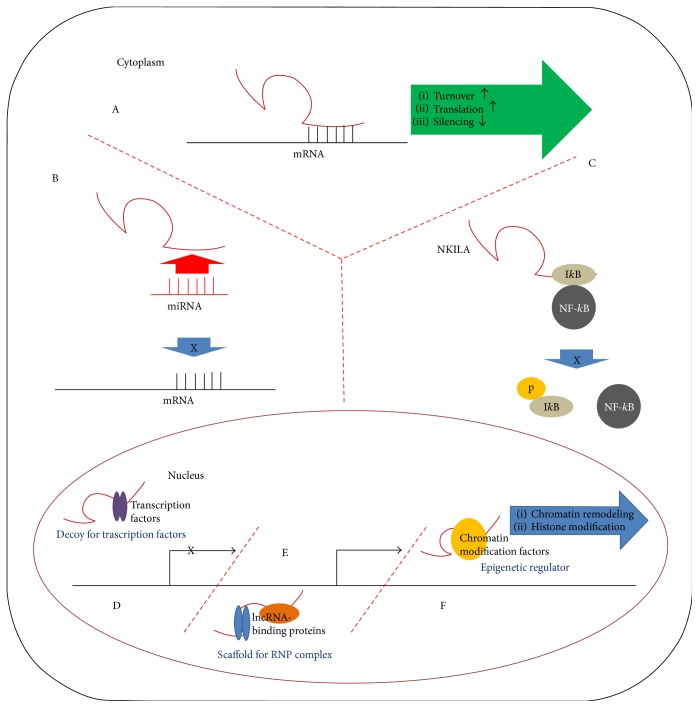
Schematic diagram of lncRNA-mediated regulatory mechanism. In cytoplasm, (A) lncRNAs can regulate turnover, translation, and silencing of partially complementary mRNAs. (B) lncRNAs can act as a miRNA sponge, reducing miRNA availability. (C) lncRNAs can modulate signaling pathways by interacting with signaling molecules. In nucleus, (D) nuclear lncRNAs can be decoys for regulatory proteins such as transcription factor. (E) Nuclear lncRNAs can serve as a scaffold for RNA-protein complex. RNP: ribonucleoprotein. (F) Nuclear lncRNAs can be an epigenetic regulator by recruiting chromatin modification factors.

LncRNAs are a class of RNAs that do not encode proteins but participate in multiple biological processes. Thanks to the advanced RNA sequencing technology, the number of sequence-verified lncRNAs is rapidly increasing [9]. The transcriptional process of lncRNAs is the same as that of protein-coding messenger RNAs (mRNAs). RNA polymerase II (PolII) transcribes lncRNAs from genomic loci, and the transcribed lncRNAs are frequently 5′ capped, spliced, and polyadenylated [10]. Except few specific characteristics of lncRNAs (lack of translated open reading frames, relatively shorter length, and poor conservation of primary sequence [11]), there is no known fundamental biochemical difference between messenger RNAs (mRNAs) and lncRNAs. A recent review authored by Quinn and Chang can provide more information on the general lifecycle of lncRNAs and known functions in depth [12]. Accumulating data indicates that lncRNAs mediate numerous regulatory processes such as imprinting genomic loci, chromosomal conformation, and allosteric regulation of enzyme activity [13, 14]. LncRNA-mediated regulatory mechanisms are diverse, and few examples of the regulatory mechanisms of lncRNAs are described in Figure 1.

In cytoplasm, lncRNAs can regulate gene expression by modulating turnover, translation, or suppression of partially complementary mRNAs [15]. It has been reported that a double-stranded structure formed by the interaction between the Alu elements of lncRNAs and the complementary Alu elements in the 3′ untranslated region of an mRNA facilitates Staufen-mediated mRNA turnover [16]. As an example of lncRNA-mediated mRNA translation, it has been reported that lncRNA containing short interspersed nuclear elements B2 (SINEB2) repeats increased ubiquitin carboxy-terminal hydrolase L1 (Uchl1) mRNA translation through association with the 5′ region [17]. LncRNAs can also block miRNA-mediated silencing of an mRNA by masking the miRNA-binding sites on a target mRNA [18]. Some lncRNAs can act as a sponge for endogenous miRNAs, neutralizing miRNA-mediated silencing of mRNAs [19, 20]. LncRNA-mediated signaling pathway modulation in cytoplasm also has been reported. According to a previous study, NF-kappaB interacting LncRNA (NKILA) inhibits NF-*κ*B signaling by masking the I*κ*B phosphorylation sites of NF-*κ*B/I*κ*B complex, stabilizing the complex [21].

Nuclear lncRNAs can act as an epigenetic regulator or a guide by recruiting chromatin modification factors to locus. For example, lncRNA PVT1 has been reported to recruit enhancer of zeste homolog 2 (EZH2, a histone modifying enzyme) to the large tumor suppressor kinase 2 (LATS2) promoter, repressing LATS2 transcription [22]. As scaffolds, nuclear lncRNAs bring together multiple proteins to form ribonucleoprotein (RNP) complexes. Such lncRNA-RNP complexes can either affect histone modifications or stabilize signaling complexes or nuclear structures [23]. Another way of nuclear lncRNA facilitating gene expression is acting as decoys. Decoy lncRNAs modulate transcription by sequestering regulatory factors such as transcription factors and catalytic proteins, rendering them less available for transcription [24]. Additionally, lncRNAs can regulate mRNA splicing process. For example, metastasis-associated lung adenocarcinoma transcript 1 (MALAT-1) regulates alternative splicing by modulating the phosphorylation of serine/arginine splicing factors [25].

## 3. Human lncRNA in Stem Cells

During the last few years, a number of good reviews on the role of lncRNAs in stem cell biology have been published [2, 26–28]. However, those previous reviews were not specific to the lncRNAs reported to exist in human. Although animal models (i.e., mouse) provide us a great deal of information that can be directly applied to human biology, not all the information obtained from animal studies can be applied to humans. In fact, studies have indicated that lncRNAs are less conserved than protein-coding genes [11, 29]. According to a previous study conducted a clone-based genome assembly of mouse, only a small portion of mouse lncRNAs had evidence of human expression [30]. This particular study indicated that only half of the mouse lncRNA sequences (1538 out of 3051 mouse lncRNAs documented) could be mapped to the human genome assembly, and furthermore, only 14% of the mouse lncRNA sequences (439 out of 3051 mouse lncRNAs documented) had evidence of orthologous transcription in human based on expressed sequence tag (EST) or cDNA. Of course, those lncRNAs lacking evidence of human orthologs may be simply unidentified in humans as yet. However, it is still worth summarizing lncRNAs identified in human stem cells considering that one of the major purposes of studying stem cell regulation is to develop more effective clinical strategies utilizing stem cells. Therefore, this review deals with the lncRNAs (1) whose regulatory function has been confirmed in human stem cells or (2) whose existence has been verified in human and the function of its orthologs from other species has been reported in stem cells.

### 3.1. Eosinophil Granule Ontogeny (EGO)

In 2007, Wagner and colleagues first identified EGO as a long noncoding RNA nested within an intron of inositol triphosphate receptor type 1 (ITPR1) by demonstrating that EGO transcript was not associated with ribosomes [31]. According to their study, the transcript level of EGO increased during interleukin-5- (IL-5-) induced eosinophil differentiation of human CD34^+^ hematopoietic stem cells from healthy donors. Silencing EGO subsequently decreased the mRNA expression of major basic protein (MBP) and eosinophil-derived neurotoxin (EDN), suggesting that EGO was required to maintain normal level of MBP and EDN during eosinophilopoiesis of umbilical cord blood CD34^+^ cells. EGO acting as a small interfering RNA (siRNA) or protecting mRNAs from degradation by forming an RNA-protein complex has been proposed as possible mechanisms.

### 3.2. Gomafu (Alias: MIAT or RNCR2)

In 2004, retinal noncoding RNA 2 (RNCR2) was first reported in the developing retina of mouse with nuclear- or perinuclear-localized expression pattern [32]. More recent study reported that nuclear-localized RNCR2 regulates retinal cell specification acting as a suppressor of differentiation into amacrine interneurons and Müller glia cells [33]. In between those two studies mentioned above, Sone and colleagues reported a noble mRNA-like noncoding gene named Gomafu (meaning “spotted pattern” in Japanese) as a neuron-specific component of the nuclear matrix [34]. They found that Gomafu shares homologous region with RNCR2 and that human homologous sequence was located in a syntenic region of chromosome 22q12. As to the role of Gomafu in stem cell biology, it has been suggested that Gomafu and Oct4 establish an autofeedback loop to maintain pluripotency of mouse embryonic stem cells (mESCs) [35]. RNAi-mediated downregulation of Gomafu led to downregulation of Oct4, along with decreased expression of Oct4-driven pluripotency markers, such as Sox2, Klf4, Gdf3, Fgf4, and Dppa3/Stella. On the other hand, RNAi specific to Gomafu increased the expression of trophoblast markers such as Cdx2, Hand1, Eomes, and Gata3 [36–38], suggesting a possible role of Gomafu as an endogenous inhibitor of differentiation along the trophoblast lineage.

Gomafu was also involved in neurogenesis and oligodendrocyte (OL) lineage specification of neural stem cells (NSCs) [39]. Gradient of sonic hedgehog (Shh) signaling promotes differentiation of nestin-positive NSC in the ventral forebrain into bipotent Nkx2.1-expressing bipotent neuronal-OL progenitor (N/OP) that can be further differentiated into GABAergic neurons (GABANs) and OLs [40]. During basic fibroblast growth factor (bFGF) and N-terminal active form of sonic hedgehog- (N-Shh-) induced N/OP differentiation of NSCs, Gomafu was downregulated but increased again in OL lineage specification and maturation [39]. However, the underlying mechanisms of Gomafu regulation or functional consequences have not been elucidated. Another alias of Gomafu is myocardial infarction-associated transcript (MIAT) [41]. A very recent study reported that knockdown of MIAT promoted osteogenic differentiation of human adipose-derived stem cells (hASCs), suggesting its role as an endogenous suppressor of osteogenic differentiation of stem cells [42]. Nevertheless, the underlying mechanisms remain unexplored.

### 3.3. Embryonic Ventral Forebrain 2 (Evf2)

Evf2 is an antisense lncRNA to distal-less homeobox 6 (Dlx-6) genes of Dlx-5/6 cluster, and it forms a complex with Dlx-2 to increase the activity of Dlx-5/6 enhancer during neurogenesis [43]. Evf2 is transcribed from the Dlx-5/6 intergenic enhancer elements ei and transcripts analysis using human EST database indicated that it is conserved in human. Although no specific function of Evf2 in stem cell has been elucidated, it has been reported that its expression increased during GABAN differentiation of N/OP [39].

### 3.4. Metastasis-Associated Lung Adenocarcinoma Transcript 1 (MALAT-1)

MALAT-1 is an oncogenic lncRNA conserved across several species including human and highly expressed in lung, pancreas, and non-small-cell lung cancer [44]. It was first reported in cancer, and accumulating data also indicate it plays significant roles in cancer stem cell biology. MALAT-1 has been reported to activate the transcription of latent-transforming growth factor beta-binding protein 3 (LTBP3), a transforming growth factor beta (TGF-*β*) bioactivity-regulating gene [45], by recruiting transcription factor Sp1 to the LTBP3 promoter in MSCs from myeloma patients [46]. The oncogenic role of MALAT-1 may be partially mediated by TGF-*β* production because TGF-*β* can promote tumor cell growth by triggering interleukin-6 (IL-6) and vascular endothelial growth factor (VEGF) production [47].

According to another study using human glioma stem cell line SHG139S, downregulation of MALAT-1 suppressed the expression of stemness markers such as Sox2 and Nestin, while it increased the proliferation of SHG139S by activating ERK/MAPK signaling [48]. Furthermore, MALAT-1 increased the proportion of chemotherapy-resistant pancreatic cancer stem cells with enhanced self-renewing capacity, and this was related to the increased expression of Sox2 [49]. A more recent study demonstrated that MALAT-1, with another lncRNA highly upregulated in liver cancer (HULC) [50], increased the expression of telomere repeat-binding factor 2 (TRF2) and accelerated liver cancer stem cell growth [51]. Such stemness and proliferation regulating role of MALAT-1 are reported in non-cancer stem cells such as iPSCs [52] and hematopoietic cells [53], as well.

### 3.5. H19

lncRNA H19 is the first lncRNA discovered, and it is paternally imprinted [54]. Human H19 is mapped to the H19-insulin-like growth factor 2 (IGF2) loci of chromosome 11p15.5. H19 transcribes a 2.3 kb lncRNA composed of 5 exons. From the first exon, H19 transcript produces the oncomir miR-675-5p and miR-675-3p [55]. MiR-675 is known to be expressed exclusively in the gestational placenta inhibiting placental growth [56]. The expression of miR-675 and H19 has been verified in human cells [57, 58]. The first evidence that H19 is involved in embryonic stem cell differentiation was reported in 1991. Poirier and colleagues demonstrated that H19 expression was activated during early murine embryogenesis [59]. Recently, the role of H19-IGF2 locus in adult hematopoietic stem cell (HSC) quiescence was reported. Maternal-imprinted H19-derived miR-675 suppresses IGF2-IGFR1 signaling pathway leading to Foxo3-mediated cell cycle arrest. This causes adult HSC quiescence which is required for long-term maintenance of HSC [60].

### 3.6. HOX Antisense Intergenic RNA Myeloid 1 (HOTAIRM1)

HOTAIRM1 was discovered as a myeloid-specific intergenic lncRNA of human HOXA1 and HOXA2 [61]. In their study, Zhang and colleagues reported that HOTAIRM1 was upregulated during granulocyte differentiation of human HSCs in a myeloid lineage-specific manner. Furthermore, knockdown of HOTAIRM1 attenuated the expression of CD11b and CD18, well known myeloid cell markers [62], suggesting HOTAIRM1 is an important mediator of myeloid cell differentiation.

### 3.7. Maternally Expressed Genes 3 (MEG3)

Another human-imprinted gene MEG3 is located in the delta-like homolog 1 gene and type III iodothyronine deiodinase (DLK1-DIO3) locus on human chromosome 14q [63]. MEG3 is expressed in normal tissues but downregulated by aberrant DNA methylation in human cancers implying its role as a tumor suppressor [64, 65]. Genomic imprinting of MEG3 is unstable in human ESCs [66], as well as in iPSCs [67]. According to the study conducted by Mo and colleagues, human ESCs with low MEG3 expression level (designated as MEG3-OFF) also showed significantly low expressions of DLK1-DIO3 locus-derived noncoding RNAs, including MEG8, miR-127, miR-376, miR-494, miR-495, miR-496, and miR-154, compared to its counterpart MEG3-ON [68]. Further, they demonstrated that MEG3-OFF led to suppressed expression of neural lineage markers such as PAX6, RTN1, and Sox11, suggesting its role as a positive regulator of neuronal differentiation.

Another known function of MEG3 is to recruit polycomb repressive complex 2 (PRC2) to chromatin to maintain transcriptional repression of lineage-specific genes during development. PRC2 is responsible for the di- and trimethylation of lysine 27 in histone H3 (H3K27me2/3) [69], which is one of the important characteristics of inactive heterochromatin [70]. MEG3 recruits PRC2 to chromatin via interaction with jumonji family, ARID domain-containing protein 2 (JARID2) [71]. Chromatin-bound JARID2 further interacts with MEG3 forming a scaffold for maximum PRC2 recruitment. Subsequently, PRC2 is recruited and assembled on a specific location of chromatin, resulting in increased H3K27me3 during ESC differentiation. This suggests that MEG3 is an important factor in epigenetic regulation of lineage-specific genes during ESC differentiation.

Additionally, MEG3 has been implicated in osteogenic differentiation of stem cells, but the results have been inconsistent depending on the source of stem cells. First, MEG3 has been reported to promote osteogenic differentiation of MSCs from multiple myeloma patients by releasing sex-determining region Y box 2- (SOX2-) mediated transcriptional suppression of bone morphogenetic protein 4 (BMP4) promoter [72]. However, few years later, the antiosteogenic effect of MEG3 in bone marrow MSCs of postmenopausal osteoporosis patient by increasing the expression of miR-133a-3p was reported [73]. Although the mechanism of such discrepancy still remains unclear, disease type-dependent effect is suspected.

### 3.8. Nuclear-Enriched Abundant Transcript 1 (NEAT1)

As the full name indicates, NEAT1 is frequently observed in nuclei, especially in the subnuclear body called paraspeckles [74]. Paraspeckles were first identified as a distinct form of nuclear structure different from the nuclear speckles that are enriched in splicing factors [75]. Paraspeckles regulate the expression of genes in differentiated cells by nuclear retention of mRNAs. The formation of paraspeckles around NEAT1 has been reported by several groups [74, 76, 77]. Paraspeckles are only observed in mammalian cells, including primary cell lines and embryonic fibroblasts [78, 79]. However, paraspeckles are not present in undifferentiated human embryonic stem cells but are induced upon differentiation [76]. Other than the role as a mediator of RNA retention, NEAT1 has been implicated in the adipogenic differentiation of ADSCs into adipocytes. Mature miR-140 interacts with NEAT1 in the nucleus, and this subsequently increases NEAT1 expression leading to adipogenesis [80]. However, they only demonstrated that miR-140-mediated NEAT1 expression is required for adipogenesis, without providing the underlying mechanism of how the increased NEAT1 contributed to adipogenesis.

### 3.9. LincRNA-Regulator of Reprogramming (LincRNA-RoR)

LincRNA-RoR was first identified as an lncRNA enriched in human iPSCs and suspected as a modulator of chromatin complexes to regulate pluripotent cell-specific epigenetic architecture [81]. Another study demonstrated that lincRNA-RoR functioned as a miRNA sponge for the miRNAs targeting embryonic stem cell-enriched transcription factors such as Oct4, Sox2, and Nanog. Consequently, lincRNA-RoR prevented miRNA-mediated suppression of these transcription factors, enhancing the self-renewal ability of human ESCs [82].

### 3.10. NoRC-Associated RNA (Promoter-Associated RNA, pRNA)

Promoter-associated RNAs were described *in vitro* in human [83]. Maturation of 250~300 nucleotide long pRNA is achieved by processing 2 kb long intergenic spacer rRNA (IGS-rRNA) [84]. Regarding the role of pRNA in stem cells, it has been reported that mature pRNA-mediated association between the nucleolar transcription terminator factor 1 (TTF1) and TTF1-interacting protein 5 (TIP5) was prerequisite to the generation of heterochromatic rDNA required for exit from pluripotency during ESC differentiation [85]. This indicated that mature pRNA may function as an initiator of ESC differentiation.

### 3.11. Antisense to Nitric Oxide Synthase 2A (Anti-NOS2A)

Anti-NOS2A shares high antisense homology (approximately 80%) to the corresponding regions of NOS2A gene. It is speculated that the anti-NOS2A is a result of gene duplication followed by an internal DNA inversion [86]. The anti-NOS2A functions as a natural antisense transcript that regulates NOS gene expression. In mammalian brain, upregulation of NOS2A has been associated with neurogenesis suggesting that NOS-mediated endogenous NO production is important in neuronal differentiation [87]. In line with such findings, the expression of anti-NOS2A significantly decreased in neurospheres compared to that in undifferentiated human ESCs, while the expression of NOS2A showed an opposite pattern [86]. This indicated that anti-NOS2A may act as a neuronal differentiation suppressor in ESCs.

### 3.12. Small Nucleolar RNA Host Gene 3 and 1 (SNHG3 and SNHG1)

Pertaining to the role of SNHG3 in stem cells, it has been reported that SNHG3 is one of the 26 lncRNAs that are required to maintain the pluripotency program of ESCs [88]. More specifically, knockdown of SNHG3 in ESCs significantly decreased the expressions of pluripotency markers including Oct4, Sox2, Nanog, Klf4, and Zfp43. Furthermore, the expression level of SNHG3 was downregulated during retinoic acid-induced differentiation of ESC, indicating that SNHG3 was one of the lncRNAs that regulate pluripotency program of ESCs. As the underlying mechanism, an ESC state controlling circuitry was proposed where ESC-specific transcription factors (e.g., Oct4, Sox2, and Nanog) derive the transcription of SNHG3, and the produced SNHG3 forms an RNA-protein complex that represses cell type-specific gene expression program. As to the role of SNHG1, it has been reported that the expression of SNHG1 significantly increased during lineage restriction of NSCs (neural stem cells) into N/OPs (bipotent neuronal/oligodendrocyte progenitors) [39]. Recent studies indicated that both SNHG1 and SNHG3 were highly correlated with poor prognosis in cancer patients [89, 90].

### 3.13. SOX2 Overlapping Transcript (SOX2OT)

SOX2 is a HMG-box transcription factor contributes to the maintenance of pluripotency of undifferentiated ESCs [91]. In humans, approximately 700 kb long SOX2OT gene is mapped to chromosome 3q26.3 locus and the SOX2 gene is embedded in the intronic region of SOX2OT; therefore, it gets its name [92]. Concomitant upregulation of SOX2 and SOX2OT in ESCs, which decreased upon differentiation, has been reported [93], and SOX2OT functioning as an enhancer to the transcription of SOX2 was postulated as the possible underlying mechanism [94]. These reports indicate that SOX2OT modulates pluripotency of stem cells via regulation of SOX2.

### 3.14. VLDLR Antisense RNA1 (LincRNA-VLDLR)

LincRNA-VLDLR was first identified as one of the lncRNAs significantly upregulated in human iPSCs and ESCs [81]. According to that particular study, pluripotency-regulating transcription factors such as Oct4, Sox2, and Nanog colocalized on the promoter of the lincRNA-VLDLR, suggesting pluripotency-regulating transcription factors induce the expression of lincRNA-VLDLR, and in turn, lincRNA-VLDLR regulates the maintenance of pluripotency. However, no specific target of the lincRNA-VLDLR has been identified regarding the maintenance of stem cell pluripotency [95].

### 3.15. Cardiac Mesoderm Enhancer-Associated Noncoding RNA (CARMEN)

CARMEN was first characterized by Ounzain and colleagues as an lncRNA associated with human cardiac-specific enhancer [96]. Enhancers are an important regulatory sequences within the genome that integrates temporal, spatial, and environmental cues to regulate gene expression [97]. Enhancer-associated noncoding RNAs play important roles in heart development and disease [98]. CARMEN, as one of such enhancer-associated noncoding RNAs, is crucial for cardiac specification and differentiation of human cardiac progenitor cells (CPCs), and this was evidenced by the observation that knockdown of CARMEN inhibited cardiac specification and differentiation of human CPCs [96].

### 3.16. Rhabdomyosarcoma 2-Associated Transcript (RMST)

RMST was first identified as an lncRNA significantly upregulated upon neuronal differentiation of human ESCs [99]. A year later, the same group elucidated the underlying mechanism of RMST in neuronal differentiation. The expression of RMST was suppressed by RE1-silencing transcription factor (REST), a master negative regulator of neuronal differentiation [100], in undifferentiated human ESCs but increased upon neuronal differentiation of human ESCs. Furthermore, nuclear localized RMST during neuronal differentiation bound to hnRNPA2/B1, a ubiquitous RNA-binding protein [101], and Sox2. Binding of RMST to Sox2 promoted Sox2 binding at the promoters of neurogenic genes, such as achaete-scute homolog 1 (ASCL1 [102]) and distal-less homeobox 1 (DLX-1 [103]), driving neuronal differentiation of human ESCs [104].

### 3.17. Human Endogenous Retrovirus Subfamily H (HERVH)

HERVH is a transposable element preferentially expressed in human ESCs. It has been reported that the long terminal repeats of HERVH regulated the expression of neighboring pluripotency marker genes by binding to both Oct4 and coactivators such as p300 in human ESCs [105].

## 4. Possible Clinical Applications of the lncRNAs in Stem Cells

The research on roles of lncRNAs in stem cell biology is still in its infancy. Furthermore, only few cell therapies using stem cells have demonstrated satisfying clinical benefit to warrant their clinical use [106]. In other words, both the research on the lncRNAs in stem cells and the cell therapies using stem cells are far from being completed. Therefore, it is very difficult to discuss the clinical implications of lncRNAs in stem cells in depth. However, approximately half million single nucleotide polymorphisms (SNPs) in more than 30,000 human lncRNAs have been identified already [107], and this strongly suggests high possibility of dysregulated lncRNAs even in stem cells. It has been demonstrated that dysregulation of lncRNAs contributes to numerous human diseases, and even very simple mutations such as SNPs can have tremendous consequences in terms of lncRNA structure and function [108].

Assuming one of the major purposes of studying the stem cell biology is to design more effective cell therapy approaches, dysregulated lncRNAs in stem cells can be a selection marker for screening adequate stem cells for transplantation. Another possible application of lncRNAs in stem cells is to use lncRNAs as a therapeutic target for reinforcing stem cell function. For example, gene-editing technology can be utilized to facilitate in situ differentiation of stem cells into desired lineage of cells by editing lncRNAs that promote or suppress differentiation of stem cells. Additionally, finding and using small molecule that induces or inhibits specific lncRNAs may serve as a powerful tool to enhancing the functionality of stem cells. However, these therapeutic applications of lncRNAs in stem cells can be realized only when the regulatory mechanisms of lncRNAs in stem cells are sufficiently elucidated.

## 5. Conclusions and Perspectives

An increasing number of studies have provided evidence that lncRNAs are important regulators of the differentiation and pluripotency maintenance of stem cells. Studies have demonstrated that lncRNAs modulate stem cell biology by interacting with essential transcription factors responsible for maintaining pluripotency or regulating differentiation. It is clear that lncRNAs play critical roles in different types of human stem cells with diverse mechanisms. Furthermore, the existence of dysregulated lncRNAs adds a new layer of complexity to the molecular mechanisms of human disease. Despite recent progress in this field, we still have a long way to fully comprehend the roles of these important regulators in human stem cells. Therefore, further studies are strongly required, and it is expected that future study of lncRNAs in stem cells will provide us new therapeutic targets for the prevention and treatment of human disease.
